# Editorial: Intervening in structural determinants: the role of language and narrative in enacting power to define issues and control resource distribution

**DOI:** 10.3389/fpubh.2024.1381416

**Published:** 2024-05-21

**Authors:** Monica L. Wendel, Tasha L. Golden, Maury Nation, Brandy N. Kelly Pryor

**Affiliations:** ^1^Department of Health Promotion and Behavioral Sciences, University of Louisville School of Public Health and Information Sciences, Louisville, KY, United States; ^2^Arts + Mind Lab, Johns Hopkins Medicine, Baltimore, MD, United States; ^3^College of Education and Human Development, Vanderbilt University, Nashville, TN, United States

**Keywords:** structural determinants, language, narrative, power, equity

Public health developed in response to infectious disease prevention and control and was thus based on an individualistic biomedical model of health (1). Interrupting one or more connections in the disease triangle proved effective for communicable diseases resulting in health education, better sanitation, and vaccinations (2). As the leading causes of death shifted to chronic diseases, however, the biomedical model was not similarly effective because the diseases could not be reduced to just a triangle—there was not one pathogen causing a disease. The field thus experienced a significant paradigm shift in the 1990s with the widespread adoption of the social ecological model, which incorporated interactions between individuals and their social environments into our understanding of how health is produced and maintained (3).

“Social determinants of health”—those factors and experiences that significantly affect health yet lie beyond any single individuals' ability to control—became a framework for acknowledging the influence of the contexts in which people live, work, worship, learn, play, and age (4). Social determinants of health became the mechanism for discussing how poverty, unemployment, racism, and other experiences in the social environment affected health outcomes. Given a lack of individual control, it became clearer that advancing health equity would require a greater focus on addressing those social determinants. In the past decade, many public health and social scientists have gone beyond social determinants to recognize and name the role of *structural* determinants of health—those factors that are the “determinants of the determinants” (5). These include not only societal structures such as laws, policies, and practices that create or perpetuate the social determinants, but also the ideologies and systems of power by which these structures have been created and maintained, such as settler colonialism, white supremacy, heteropatriarchy, and racial capitalism (6). Research on chronic diseases suggests that public health must understand and disrupt these social and structural determinants if we hope to replicate the successes experienced with prevention of infectious diseases. Such structural determinants are rooted in the distribution and reinforcement of power: determining who has it and how it is wielded.

As contemporary global events continuously highlight social and health inequities in communities, municipalities, and broader society, increased attention must be paid to the role of *power* in creating systems, policies, and practices that benefit some groups while enacting violence on others. Oppressive ideologies do not emerge *ex nihilo* but are rather enacted by those who have accumulated or maintained power to control the distribution and flow of various kinds of resources and capital. In other words, an understanding of how power is exercised in the creation of the social and structural determinants is a key to the development of effective public health interventions to improve population health.

While multiple health and social science disciplines have been engaged in attempting to understand the role of power in shaping society, recognizing and acknowledging structural determinants is not enough. If these structures established by power are the root causes of inequity, they are public health issues, and influencing them has potential to radically change myriad downstream outcomes that are disparately and inequitably experienced. Thus, we must identify key leverage points to change them.

One critical leverage point across structures and systems is *narrative*—the language and stories that are created, elevated, heard, believed, and acted upon. Within our systems, narratives are a mechanism for producing and enacting power. The words used to define or describe specific public health issues inherently point us to specific solutions and justify allocation (or withdrawal) of resources. Those words and their narratives can help advance health equity, *or* they can reproduce the social inequity and health disparities faced by structurally marginalized communities. For example, language in the field is being intentionally shifted to be more person-centered at the individual level (e.g., “minoritized populations” vs. “minorities;” “people experiencing homelessness” vs. “the homeless”), and more focused on who is responsible for issues at higher ecological levels (e.g., “inequities” vs. “disparities;” “structurally marginalized” vs. “disadvantaged” or “at-risk”). Far from mere semantics, these shifts in language result in changes to how a given issue or population is understood, what kinds of solutions are generated, and how they are enacted.

The Research Topic collection in this issue offers many more examples of the power of language and narrative to affect health at structural levels. It consists of six original articles that advance our understanding of the impact of language and narrative as an enactment of power—a critical force in public health and health equity. Bowleg unpacks the power of language and public health discourses, including how the white racial frame shapes health discourses and thus understandings about disparities and inequities. She also discusses the “cost and consequences of the white racial frame” and calls for increased attention to power, language, and discourse and adoption of “counter and critical theoretical frames to inform discourses, and in turn research and political advocacy to advance health equity in the U.S.” In their article “Stories of self, us, and now: Narrative and power for health equity in grassroots community organizing,” Haapanen et al. draw upon interview data with community leaders in two cities to discuss processes by which public narratives are constructed and changed. Their findings indicate that “the process of narrative change is deeply relational and dynamic, a finding that challenges assumptions of some programmatic or top-down approaches to narrative intervention.”

Two of the articles consider the importance of narrative in defining constructs within violence prevention efforts, naming the structural violence of dominant narratives and the logics they uphold. Buggs et al.'s manuscript, “*Voicing narratives of structural violence in interpersonal firearm violence research and prevention in the United States*,” calls attention to the lack of focus on structural violence in narratives about firearm violence. They argue that research that fails to explicitly address the narratives linking structural violence with interpersonal violence “invite, perhaps even necessitate, the application of racialized schemas and mental heuristics” in violence research “that inevitably push the mainstream narrative toward [the] individual level…pathologizing environmentally responsive survival strategies and reinforcing status quo approaches.” The authors also emphasize the importance of including assets-based language in narratives around violence prevention, calling attention to peace-making and human agency in creating a healthy and safe community. In their article “Relationships, resources, and political empowerment: Community violence intervention strategies that contest the logics of policing and incarceration,” Dawson et al. demonstrate how the logics of policing and incarceration actually perpetuate violence and increase harm, while “elevating the language, narratives, and values of outreach-based community violence intervention and prevention can transform our responses to violence, interrupt cycles of harm, and foster safer communities.”

Finally, two articles home in on specific narrative-based interventions at different levels of social ecology. Haworth-Brockman et al.'s article, “*Saying it out loud: Explicit equity prompts for public health organization resilience*,” discuss the importance of making a focus on health equity explicit, visible, and sustainable so that the efforts do not get sidelined or forgotten in the face of other urgent issues. They conducted training for public health professionals working in emergency preparedness to practice this equity focus and help move their commitment to health equity “from theory to true preparedness and resilience.” Schillinger et al. present findings from their study of the “Survival Pending Revolution” campaign to increase COVID-19 vaccination among young adults of color. The campaign engaged members of structurally marginalized populations “as creators and messengers of campaigns…whose goal is to aid disparity populations in both resisting and navigating systems that continue to locate them on the margins of society.” Like Haapanen et al., these young members created video poetry that invoked stories of self, stories of us, and stories of now to reflect public dialogue on rationales for vaccination within the historical and racialized context of their communities. Their narratives resonated with peers and suggested more opportunities to inform, create, and elevate new narratives.

In analyzing how narrative and language enact power to construct and reinforce structural determinants of health, the authors of this Research Topic illuminate potential means by which power—often constructed and reinforced via narrative—can be challenged or generated through an alternative collective process. Thus, this Research Topic is an important contribution in the continued development of the public health knowledge base related to how we might affect social and structural factors to prevent chronic disease and the disproportionate impact on marginalized populations. By highlighting new strategies identifying and utilizing public narratives to understand and intervene in social problems, our intention is that this Research Topic will prompt the development of theory and research on how public health might leverage narrative to redistribute power and create equitable structures in our efforts to improve population health and advance equity.

## Author contributions

MW: Conceptualization, Writing – original draft. TG: Conceptualization, Writing – review & editing. MN: Writing – review & editing. BK: Writing – review & editing.

## References

[1] Golden TL Wendel ML. Public health's next step in advancing equity: re-evaluating epistemological assumptions to move social determinants from theory to practice. Front Pub Health. (2020) 8:482389. 10.3389/fpubh.2020.00131 32457863 PMC7221057

[2] Centers for Disease Control and Prevention. Ten great public health achievements–United States, 1900-1999. MMWR. (1999) 48:241–3.10220250

[3] McLeroy KR Bibeau D Steckler A Glanz K. An ecological perspective on health promotion programs. Health Educ Q. (1988) 15:351–77. 10.1177/109019818801500401 3068205

[4] Robert Wood Johnson Foundation. A New Way to Talk About the Social Determinants of Health. Princeton, NJ: Robert Wood Johnson Foundation (2010).

[5] Williams GH. The determinants of health: structure, context and agency. Sociol Health Illness. (2003) 25:131. 10.1111/1467-9566.00344 14498934

[6] Maree Stanley J. Intersectional and relational frameworks: confronting anti-Blackness, settler colonialism, and neoliberalism in US social work. J Prog Hum Serv. (2020) 31:210–25. 10.1080/10428232.2019.1703246

